# Cooperation behavior of fore‐ And hindlimbs during jumping in *Rana dybowskii* and *Xenopus laevis*

**DOI:** 10.1002/ece3.7589

**Published:** 2021-05-03

**Authors:** Mo Li, Zibo Gao, Jili Wang, Wei Song, Qingzhu Zhang, Jin Tong, Lili Ren

**Affiliations:** ^1^ College of Biological and Agricultural Engineering Jilin University Changchun China; ^2^ The Key Laboratory of Bionic Engineering Ministry of Education Jilin University Changchun China; ^3^ School of Mechanical and Aerospace Engineering Jilin University Changchun China; ^4^ School of Engineering Huzhou University Huzhou China

**Keywords:** Fore‐ and hindlimbs, function, kinematics, *Rana dybowskii*, short jump, *Xenopus laevis*

## Abstract

Frogs are characterized by their outstanding jumping ability, depending on the rapid extension of hindlimbs to propel their bodies into air. A typical jumping cycle could be broken into four phases: preparation, takeoff, flight, and landing. Considerable research has been performed to discuss the function of hindlimbs of frogs during takeoff phase, whereas the literature of limbs' motion in jumping between different species was limited. To profile the evolution of locomotion in anurans, it is necessary to investigate on the motion of fore‐ and hindlimbs of frogs within different taxa. In this work, we put forward a detailed description of jumping behavior of two frog species, *Rana dybowskii* and *Xenopus laevis*. High‐speed cameras were used to explore the movement of different joints in fore‐ and hindlimbs of these two animals, and kinematic analysis was operated to identify both homologous behaviors and significant differences between them. We found that the *Rana dybowskii's* fore‐ and hindlimbs had good cooperation during jumping, while the *Xenopus laevis*' uncooperative behavior in limbs may give a functional explanation for the deficiency in terrestrial jumping; besides, the *R*. *dybowskii's* landing followed the “hands‐belly‐feet slap” strategy, and *Xenopus laevis* had clumsy landing with “belly‐flops” sequence. The result gained here clarifies the cooperation behavior of anuran limbs and may supply a new insight into our understanding of the anuran's evolution.

## Graphical abstract

In this work, we put forward a detailed comparative description of jumping behavior between two frog species, Rana dybowskii and Xenopus laevis. High‐speed cameras were used to explore the movement of different joints in fore‐ and hindlimbs of these two animals, and kinematic analysis was operated to identify both homologous behaviors and significant differences between them. We found that the Rana dybowskii's fore‐ and hindlimbs had good cooperation during jumping while the Xenopus laevis' uncooperative behavior in limbs may give a functional explanation for the deficiency in terrestrial jumping; besides, the R. dybowskii's landing followed the "hands‐belly‐feet slap" strategy, and Xenopus laevis had clumsy landing with "belly‐flops" sequence. The result gained here clarifies the cooperation behavior of anuran limbs and may supply a new insight into our understanding of the anuran's evolution.

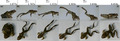

## 1 INTRODUCTION

Animals modulate their locomotion behavior to adapt the environment. Frogs have experienced a remarkable adaptive radiation since diverging from a common ancestor prior to the Triassic (Anderson et al., 2008; Essner et al., 2010; Marjanović & Laurin, 2007). Frogs’ jump is assumed to evolve from a tetrapod trotting gait, and it becomes a significant locomotion manner (Handrigan & Wassersug, 2007; Reilly & Jorgensen, 2011; Reilly et al., 2016) which gives a great contribution to their successful evolution. Some literature assumed that all anurans jump in a similar manner, while others argued that terrestrial jumping was the primitive locomotion mode from which all other locomotion modes evolved (Jenkins & Shubin, 1998; Přikryl et al., 2009; Shubin & Jenkins, 1995). Most anurans use their hindlimbs to generate propulsive force during both jumping and swimming. The same apparatus, the legs, is used to perform the same task, but in two different media (Nauwelaerts & Aerts, 2006).

Generally, frogs’ jump can be divided into four phases: preparation, takeoff, flight, and landing. The first phase begins with a crouched stationary position, preparing for the takeoff phase; the takeoff phase of jumping involves rapid hindlimbs extension accompanied by loss of forelimbs contact; the flight phase is characterized by midair body and limbs rotations in preparation for landing; landing begins when forelimbs or body touch the ground, and then, the flexed hindlimbs rotate into position beneath the body, enabling the rapid preparation of another takeoff (Azizi et al., 2014; Cox & Gillis., 2016, 2017, 2020). To escape from external danger optimally, all phases should be in short duration.

As a dominant locomotion behavior, frogs' jump has aroused a lot of attention by scholars (Akella & Gillis, 2011; Astley et al., 2013, 2015; Astley & Roberts, 2014; Astley & Thomas, 2012; Azizi & Roberts, 2010; Gillis & Biewener, 2000; Richards et al., 2017; Wang et al., 2014). Most of researches focus on the physiology, biomechanics, muscle function of the hindlimbs during takeoff, since jump mainly relies on the mechanical force and elastic energy produced by the tendon attached to the hindlimbs muscle (Abdala et al., 2018; Azizi, 2014; Kuan et al., 2017; Nauwelaerts & Aerts, 2006; Roberts & Marsh, 2003). However, the cooperation behavior and function of fore‐ and hindlimbs of different anuran phylogenies during jumping have been rarely discussed.

*Rana dybowskii* (the Chinese brown frog) is a highly specialized jumping species which takes most of its time on land. *Xenopus laevis* (the African clawed frog) is typical aquatic, and it can only take short jumps on land temporally (Herrel et al., 2014). We hypothesize that *R.dybowskii* and *X*. *laevis* have different motion mechanism during jumping, so the goal of this study is to explore the motion mechanism of these two frogs with different specialty. In the present work, we examined the kinematic features in short jumps of the two model frogs. A detailed investigation of movement on different joints of two frog species was operated using image tracking technology, and kinematic analysis was used to identify and describe the function of fore‐ and hindlimbs during jumping. The difference on joint kinematics of fore‐ and hindlimbs could reveal the diversity on motion strategy of different frogs in locomotion. Moreover, this paper may be able to shed more light on the design of frog‐inspired robot and also broaden our understanding of the anuran's evolution.

## 2 MATERIALS AND METHODS

### 2.1 Animals

Six adult *R. dybowskii* and *X*. *laevis* (unknown sex) were obtained from a commercial supplier and housed in groups of two to four in large plastic containers in a temperature‐controlled room set at 19°C–26°C and 25%–60% relative humidity with a 12 hr light:12 hr dark cycle. The animals were fed a diet of crickets several times a week, and water was always available. The body length (BL) of frog samples was defined as snout‐vent length (SVL), and it was measured to the nearest millimeter using a mechanical caliper. All experimental work was approved by Jilin University and conformed to NH guidelines.

### 2.2 Experimental setup

To record the jump trails, two synchronized digital high‐speed video cameras were used in this work. The first camera (Olympus, i‐SPEED 3, Tokyo, Japan) filmed the jumping behavior from the side view at a frame frequency of 500 Hz (1,280 × 800 pixels). The second camera filmed the frog from above (500 Hz) to check whether the frog's trajectory was in the plane of the first camera. Both cameras ran in synchrony with the data acquisition using a common starting pulse from a manual switch. The animals were placed on a glass platform, and a multiple light‐emitting diode (LED) cold light source placed above the platform was used to supply adequate illumination for filming.

### 2.3 Date extraction

Frogs were enticed to jump by tapping behind the body. Each frog was numbered and filmed 1–3 times. There were two rules to define an acceptable trail: Firstly, the trials in which frogs both took off and landed on the recording plane of two cameras; secondly, the profiles of fore‐ and hind limbs on right side (close to the side camera) were clearly recorded. We chose 10 jump trails for each frog type and then imported the 20 video files into SIMI motion analysis software (Simi Motion 2D/3D1 7.5 software, SIMI Reality Motion Systems GmbH, Germany) for image tracking and kinematic analysis. The software allows for three‐dimensional calibration, digitization of landmarks, and calculation of the segment and joint kinematic parameters of interest, and the average measurement error of this motion tracking system was ±1.0 mm (Stoessel & Fischer, 2012). To avoid substantial error caused by a low‐resolution calibration (Longo et al., 2019), a calibration cube of eighteen retro‐reflective markers (2mm in diameter) was used to enable a definition of the three direction in space (x, y, z axes) and it was filmed first as the calibration reference in the tracking system. Then, every tenth to twentieth frames were manually digitized in each of the two planes. Data between these frames were spline interpolated. Then, Simi Motion 3D calculates 3‐D coordinates, which are required to obtain angles for kinematic parameters. Parallax was corrected by calibration of the recordings before calculation of coordinates and angles. To test for accuracy of the digitization, 20 consecutive frames during the jump start were digitized and the joint of three trials were calculated. This procedure was repeated three times for the same 20 frames. The standard deviation between the angles of each frame from the three trials was calculated. The mean of the 20SD obtained was used as a measure of accuracy. The standard deviation between the angles of the three trials was constantly less than 1deg; thus, the error of digitization is acceptable.

**FIGURE 1:**
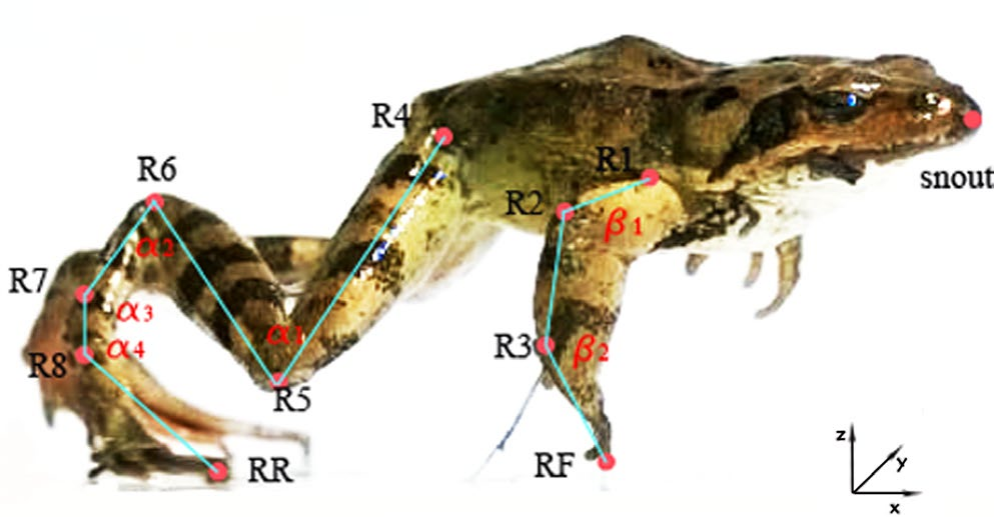
Schematic view of a frog's jump. R1‐shoulder; R2‐elbow; R3‐wrist; RF‐fore toe; R4‐hip; R5‐knee; R6‐ankle; R7‐TMT; R8‐midfoot; RR‐rear toe; **a**_1_‐knee angle; **a**_2_‐ankle angle; **a**_3_‐TMT angle; **a**_4_‐midfoot angle; β_1_‐elbow angle; β_2_‐wrist angle

**FIGURE 2:**
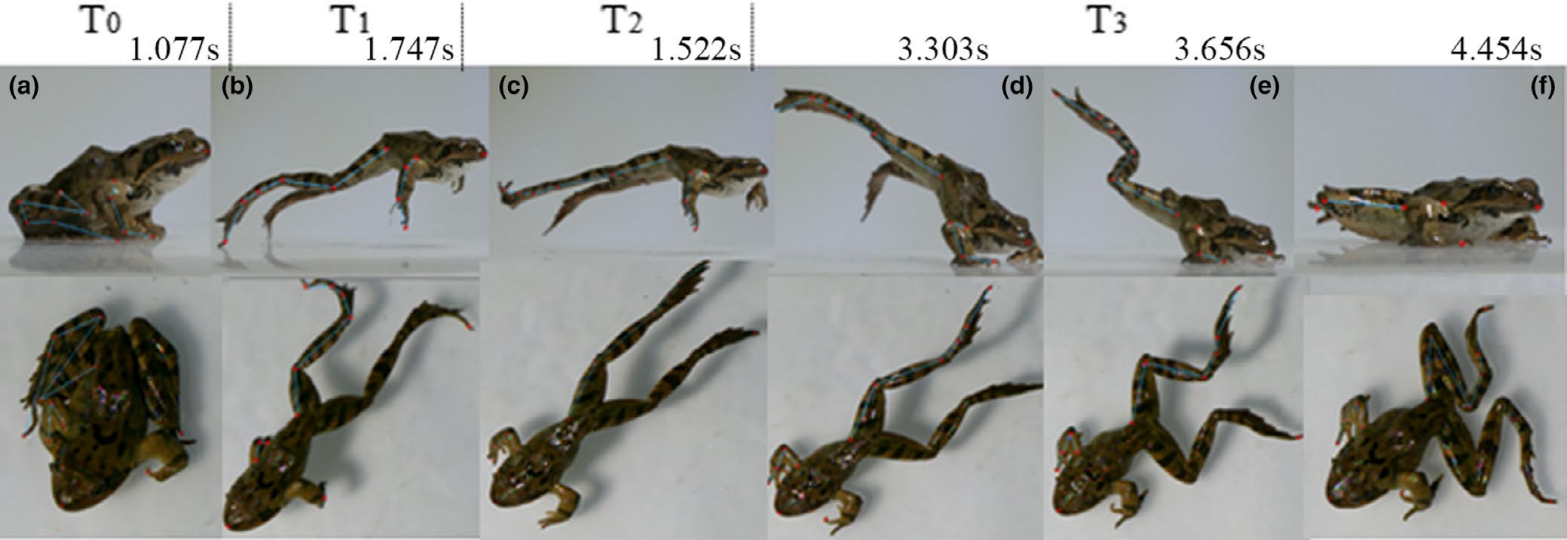
Frame sequences of a R. *dybowskii's* jump. T0‐Preparation phase; T1‐Takeoff phase; T2‐Flight phase; T3‐Landing phase; a is the head‐up moment in preparation; b is the moment hindlimbs just leave the ground; c is the initial time of flight; d is the moment forelimbs hit the ground; e is the moment the body hits the ground; f is the moment all limbs land on ground

After calibration, all video sequences were analyzed to identify the onset and end of frog's movement for each jump. All the joints in both forelimbs and hindlimbs were digitized, frame by frame, using image tracking technology in SIMI, including the shoulder (R1), elbow (R2), wrist (R3), fore toe (RF), hip (R4), knee (R5), ankle (R6), tarsometatarsal (TMT) (R7), midfoot (R8), the longest hind toe (RR), and the snout tip (Figure 1). Prior to each analysis, the 3D position trajectories of marks were filtered using a moving average over three frames. Knee angle (α1) was measured as the angle formed by the hip, knee, and ankle, and ankle angle (α2) was measured as the angle formed by the knee, ankle, and tarsometatarsus. TMT angle (α3) was measured as the angle formed by ankle and midfoot, and midfoot angle (α4) was formed by TMT and the hind toe. Elbow angle (β1) was formed by the shoulder, elbow, and wrist, and wrist angle (β2) was formed by the elbow, wrist, and fore toe. The position change of frog's head could be an obvious sign of takeoff or landing phase, so the snout tip on head is able to give us a clear picture to figure out the upper body movement. Moreover, the snout of frog is also an easy tracking mark, and jump distance could be calculated between the starting and ending positions of the snout tip. The joints velocities were calculated in SIMI Reality Motion Systems from the filtered first derivative of the 3D displacement of the marks over time. Besides, from Figure 1, the x‐axis is on the forward direction of frog's jump, z‐axis is on the upward direction, and y axis is along the lateral side. Jump is powered by the extension of hindlimbs, and the kinematic behavior of knee is capable to describe the jumping motion of hindlimbs. Additionally, the snout tip can reveal the movement of front half body. Therefore, velocity on knee (V_5_) and snout (Vs) at some key moments of different jump phases were chosen to characterize the jump kinematics in this work. These key moments included the time points at the hindlimbs left the ground, the forelimbs hit the ground, etc., as shown in Figure 2. For each video frame in every jump, α1,2,3,4 and β1,2 were defined using imaging measurement tool in SIMI and were used to quantify angular excursions; besides, the duration of each phase in every jump was calculated.

### 2.4 Statistics

All reported values represent means ±SD If more than one trial was obtained for an individual, the data were averaged to represent that individual to avoid pseudo‐replication. Correlation analysis was used to test the influence of frog species and body length on marks velocities, respectively. The statistical analyses were done in SPSS software (SPSS, Inc.).

## 3 RESULTS

The measurement result of samples' body sizes is shown in Table 1, and the mean body length of *R*. *dybowskii* was longer than *X*. *laevis* while the body weight of *R. dybowskii* was similar with *X*. *laevis*.

**TABLE 1:** Body length (BL) and body weight (BW) of each sample

	S_1_	S_2_	S_3_	S_4_	S_5_	S_6_	Means ± SD
*R. dybowskii*							
BL, mm	50	50.4	56.1	60.7	57.6	54.7	56 ± 4
BW, g	16.807	16.202	14.042	18.616	19.434	20.901	18 ± 3
*N*	1	1	2	2	2	2	
*X. laevis*							
BL, mm	44.5	48.6	47.3	45.8	47.1	44.3	46 ± 2
BW, g	14.281	18.634	18.014	16.007	20.04	17.61	17 ± 2
*N*	1	2	2	2	2	1	

Note

*N =* number of jump trails analyzed.

### 3.1 Angle motion of *Rana dybowskii* and *Xenopus laevis*

The representative short jump process of *R*. *dybowskii* is shown in Figure 2. The frog jump begins in a crouched resting position which is the preparation phase. Takeoff could be divided into two periods, first one is that hindlimbs extended to lift the body accompany with the forelimbs' lift, the next one is from forelimbs lift off until hindlimbs lift off. In the late period of flight phase, the frog's forelimbs moved forward and flexed to prepare for landing. The landing was defined from hand‐hit to the body‐hit the ground (Reilly et al., 2016). As shown in Figure 2, the whole jump behavior was divided with T0,T1,T2,T3, and the time points, a to f, were the key moments of each phase.

**FIGURE 3:**
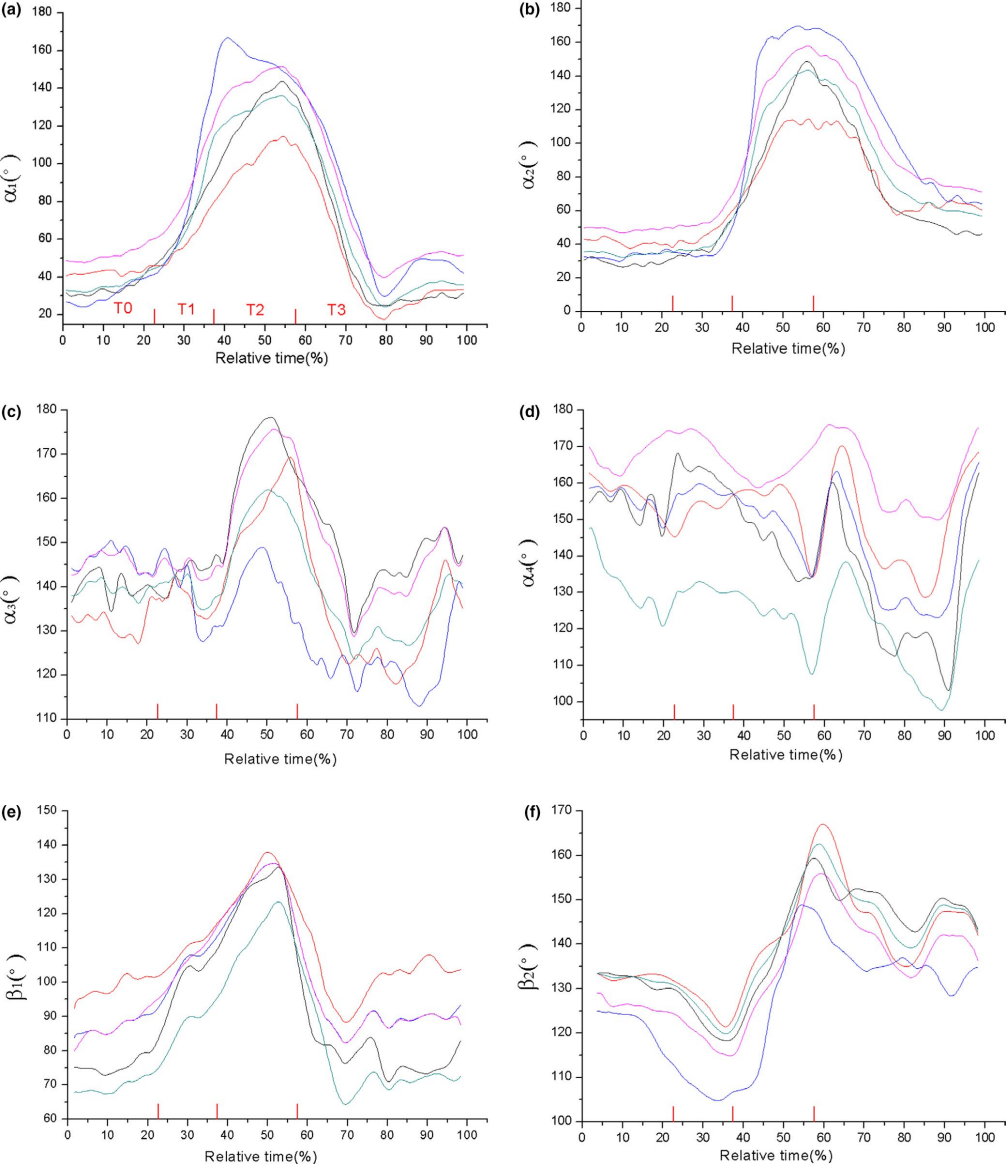
Angle motion profiles of the R. *dybowskii* ' joints during jumping, (a‐f) is the angle of knee, ankle, TMT, midfoot, elbow, and wrist, respectively

Figure 3 recorded the angle motion profiles of tracking joints during jumping trails from five samples of *R. dybowskii*. The total jumping time of each trial was set as 100%, and the jumping phases were calculated accordingly. It can be found that the knee angle **a_1_** and ankle angle **a_2_** followed a same trend in the jump process (Figure 3a and 3b). **a_1_** and **a_2_** were both around 40° in T0 phase and rapidly increased in T1, and reached to the maximum values in T2; then, they slowly dropped in T3. When the **a_2_** arrived at point b, the ankle was about to rapidly extend and leave the ground. The data of TMT angle **a_3_** were around 140° in T0, and they had great rise in T2 and then dropped quickly in T3 (Figure 3c). After the frog body hits the ground (point e in Figure 2), **a_1,_ a_2,_** and **a_3_** had a tendency to return to their initial states. The midfoot angle **a_4_** had slight changes during T0, T1, and T2, while it showed obvious fluctuations in landing phase T3 (Figure 3d). As for the forelimb joints, elbow angle β_1_ was in the range of 65°–95° in T0 (Figure 3e) and kept rising in T1 and T2, while in landing phase, they rapidly dropped to their original values. The wrist angle β_2_ (Figure 3f) firstly had a slow decrease in T1 and quickly increased in T2, and then, they gradually decreased to go back to the initial values. The change range of wrist angle β_2_ and the midfoot angle **a_4_** in four phases were smaller than other joint angles (less than 50°).

In most trails of *X. laevis* ' jump, the forelimbs could not well cooperate with the takeoff movement of hindlimbs. Although the forelimbs of *X.laevis* might successfully lift off in a few tests, the two arms showed asymmetric or unsynchronized rotation and the duration was extremely short, which makes it difficult to define the flight phase. Hence, we presumed three phases of the *X.laevis*' jump in this work.

**FIGURE 4:**
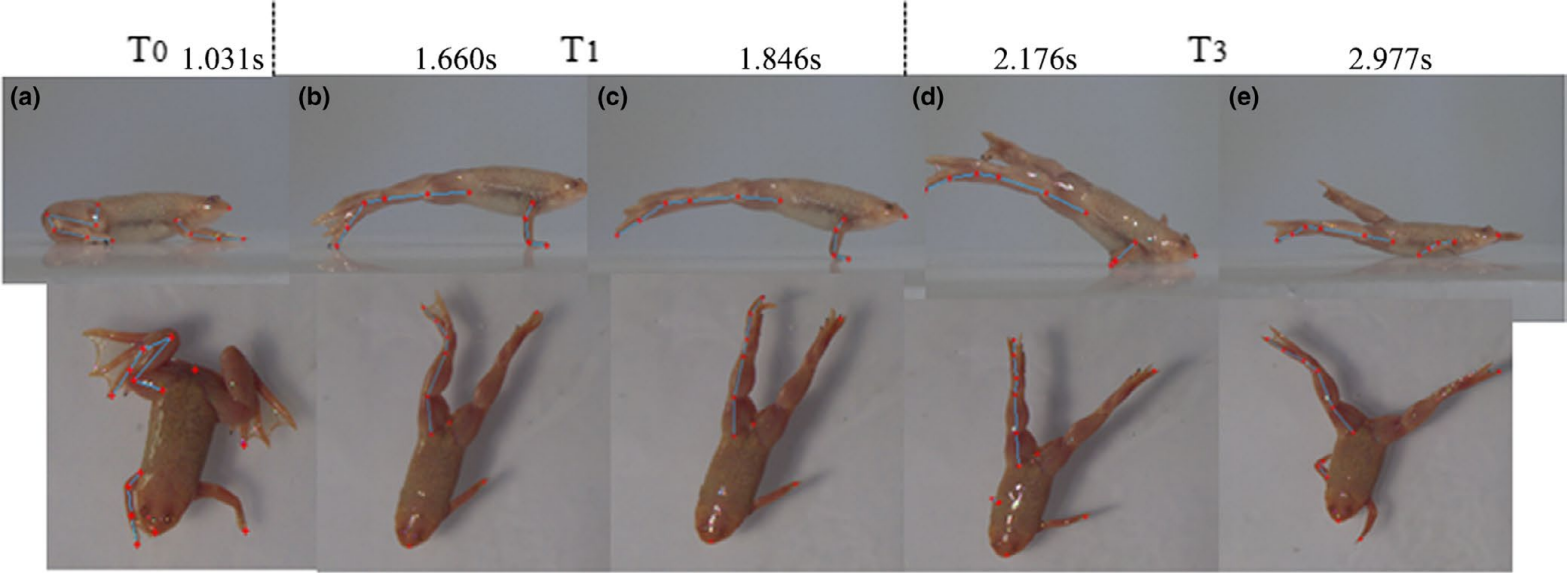
Frame sequences of a *X*.*lavis*'s jump. T0‐Preparation phase; T1‐Takeoff phase; T3‐Landing phase; a is the head‐up moment in preparation; b is the moment hindlimbs is about to leave; c is the moment hindlimbs just takeoff from the ground; d is the moment the body hits the ground; e is the moment whole body land on ground, and all limbs are about to rotate to recover preparation posture

From Figure 4, it can be seen that the *X.laevis'* jump behavior is different from the *R. dybowskii*. Its forelimbs and hindlimbs showed uncoordinated behavior. When it prepared to take off with a crouched resting position, it used forelimbs to support body and extended hindlimbs to produce propulsive force; however, the forelimbs could not lift off from the ground. Hence, in takeoff phase, only the hindlimbs lifted off and there was no clear and distinct flight phase. In a short time, the frog nosed down and the body hits the ground.

**FIGURE 5:**
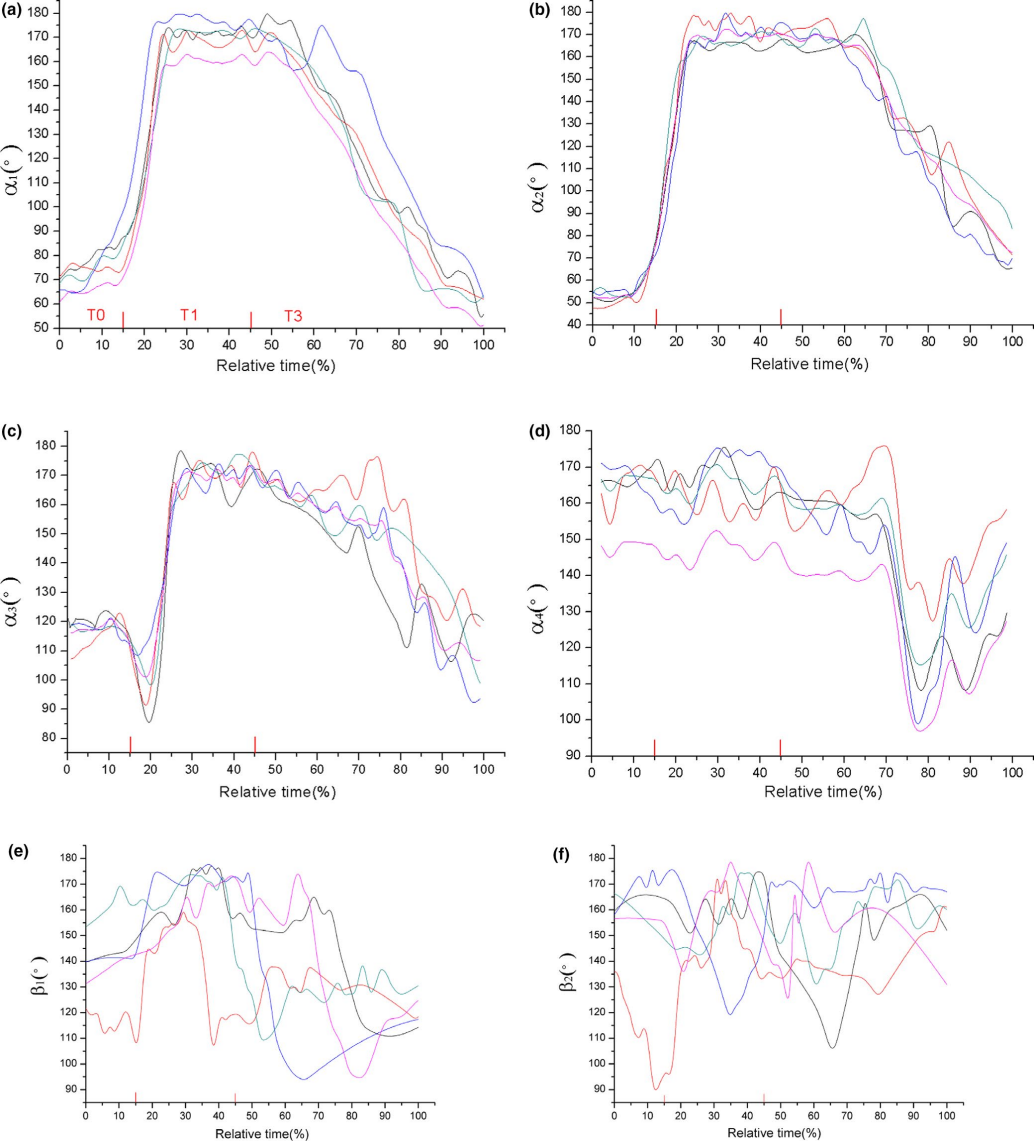
Angle motion profiles of the *X.lavis'* joints during jumping, (a–f) are the angle of knee, ankle, TMT, midfoot, elbow, and wrist,respectively

As shown in Figure 5a–5c, the knee, ankle, and TMT angle had a similar change trend. The values of **a_1_** were around 70° in T0, **a_2_** were around 50° in T0, **a_3_** were around at 120°, and the three angles greatly increased to about 170°in T1 then slowly declined in T3. The midfoot angle **a_4_** hardly changed in T0 and T1, while it went down and up in the late T3 (Figure 5d). As shown in Figure 5e, all the angles had an fast rising in T1, though there were big differences on the initial states of these elbows. However, the lines of wrist angle (β_2_) were rather messy and they had considerable differences during the whole jumping process. The wrist angles hardly showed any changing trend in common.

### 3.2 Velocities on knee and snout of *Rana dybowskii* and *Xenopus laevis*

The velocities on knee and snout of *Rana dybowskii* and *Xenopus laevis* during jumping are listed in Table 2. As for the *R*. *dybowskii*, the mean total velocity of knee (V_5_) was 0.0918 m/s at the moment of hindlimbs' takeoff (time point b) and it greatly decreased to 0.0307 m/s in flight phase (time point c) and then it went to 0.0423 m/s (time point d) and dropped to 0.0209 m/s (time point f) in landing phase. Velocity on snout tip was 0.0712 m/s at takeoff (time point b) and decreased to about 0.0303 m/s in flight phase (time point c). It rose to 0.0373 when the forelimb hit the ground (time point d) and dropped to 0.0102 m/s when the body hits the ground (time point e). It indicated that the maximum V_5_ occur at the moment of hindlimbs takeoff from the ground (the time point b), when the hindlimbs extended sufficiently to produce propulsive force. As the hindlimbs propelled the frog body into the air, velocity on snout (Vs) reached the maximum which means the body move forward. Then, the V_5_ and Vs dropped by more than half in flight phase and had small rise in point d then slowly decreased. It is worth noting that the V5 and Vs showed a similar change tendency.

**TABLE 2:** Velocities on knee and snout of *Rana dybowskii* and *Xenopus laevis* (Mean ± SD)

Time point	b	c	d	e	f
*R.dybowskii*					
Knee velocity, m/s	0.0918 ± 0.02	0.0307 ± 0.01	0.0423 ± 0.01	0.0375 ± 0.01	0.0209 ± 0.008
Snout velocity, m/s	0.0712 ± 0.02	0.0303 ± 0.01	0.0373 ± 0.01	0.0173 ± 0.004	0.0102 ± 0.003
*X.laevis*					
Knee velocity, m/s	0.0344 ± 0.008	0.0229 ± 0.003	0.0191 ± 0.006	0.0134 ± 0.004	/
Snout velocity, m/s	0.0337 ± 0.003	0.0266 ± 0.005	0.0152 ± 0.005	0.0106 ± 0.003	/

Note

For Rana, b is the moment hindlimbs just leave the ground; c is the initial time of flight; d is the moment forelimbs hit the ground; e is the moment the body hits the ground; f is the moment all limbs land on ground. For Xenopus, b is the moment hindlimbs is about to leave; c is the moment hindlimbs just takeoff from the ground; d is the moment the body hits the ground; e is the moment whole body land on ground, and all limbs are about to rotate to recover preparation posture.

For *X*. *laevis*, the V_5_ was 0.0344 m/s in takeoff phase (time point b) and it decreased to 0.0229 m/s (time point c) then gradually decreased in landing (time point d, e). For Vs, it approximated to V_5_ at time point b and then went higher than V_5_ at time point c. After that, it became lower than V_5_ during landing.

One major behavioral difference between these two animals was evident in takeoff phase. The *R. dybowskii's* forelimbs could lift accompany with the extension of hindlimbs and lift off from the ground in appropriate time, which made a good cooperation with hindlimbs. However, the *X.laevis*' forelimbs seemed to be out of rhythm of whole body, since the forelimbs could not lift as the hindlimbs take off from the ground (Figure 4). They fail to cooperate with hindlimbs, which forms an uncoordinated behavior and then makes an unsuccessful takeoff. Besides, the *R. dybowskii* protract the forelimbs to impact the ground accompany with hindlimbs retract in landing. In contrast, *X.laevis*' hindlimbs still kept protracting during landing, and the fore‐ and hind limbs begin to rotate after the body hits the ground (Figure 4). The fore‐ and hindlimbs are not able to timely adjust motion to prepare for landing, which also shows an uncoordinated movement and result in a clumsy landing.

**FIGURE 6:**
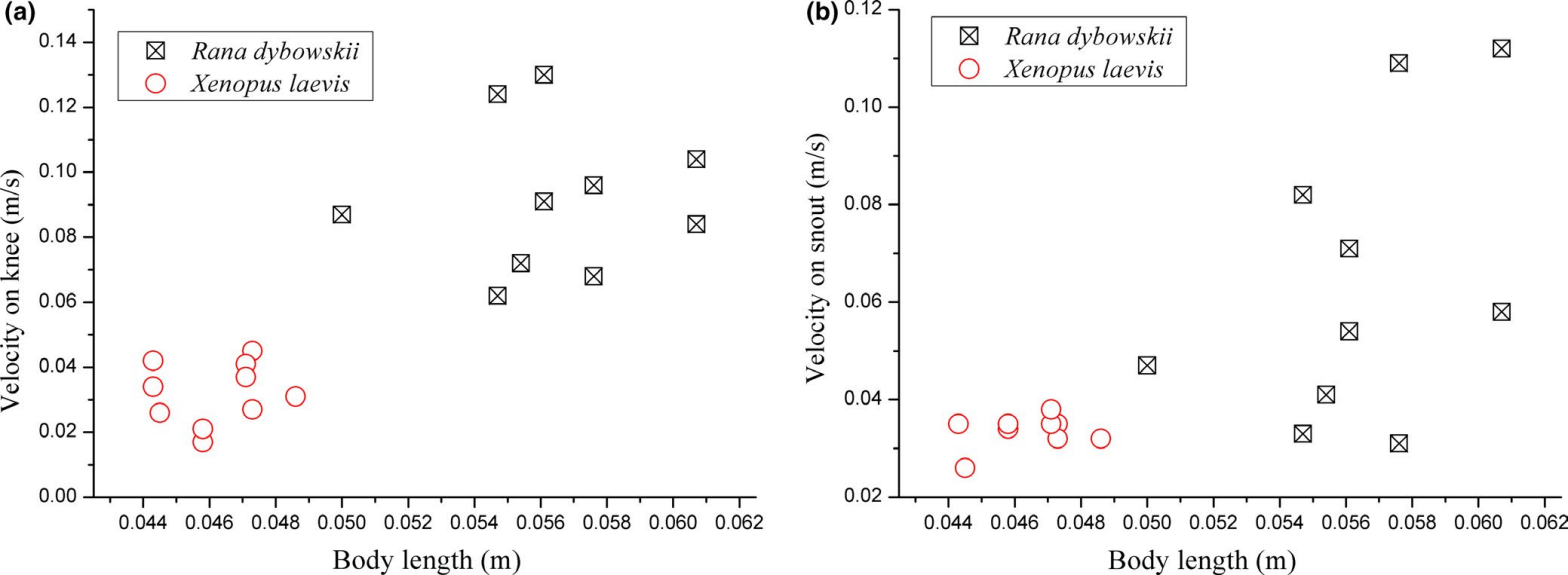
Differences on takeoff velocity of *Rana dybowskii* and *Xenopus laevis* (a) velocity on knee, V5 (b) velocity on snout, Vs

A serial of comparative data for kinematics analysis on these two animals are listed in Table 3. It was found that the takeoff duration time of *R. dybowskii* is 14.7% less than the time used by *X.laevis*. Correlation analysis result showed the species had a significant impact on velocities (sig. <0.05) while body length did not. The regression analysis on velocity at takeoff of two model frogs is shown in Figure 6. The correlation coefficient (R^2^) between takeoff velocity and body length of individual *R. dybowskii* frog was 0.007, and R^2^ of *X. laevis* was 0.037. Therefore, in this work, it was reasonable to assume that there is no relationship between the velocity and the body length of the two types of frogs.

**TABLE 3:** Results of a repeated measures analysis of jumping kinematics in two frog species (Mean ± SD)

	*R.dybowskii*	*X.laevis*
Jump distance (BL)	2–4	1.2–2
Preparation duration (s)	0.75 ± 0.3	1.14 ± 0.2
Takeoff duration (s)	0.58 ± 0.2	0.68 ± 0.2
Flight duration (s)	0.6 ± 0.4	/
Landing duration (s)	3.22 ± 0.6	4.65 ± 0.78

**FIGURE 7:**
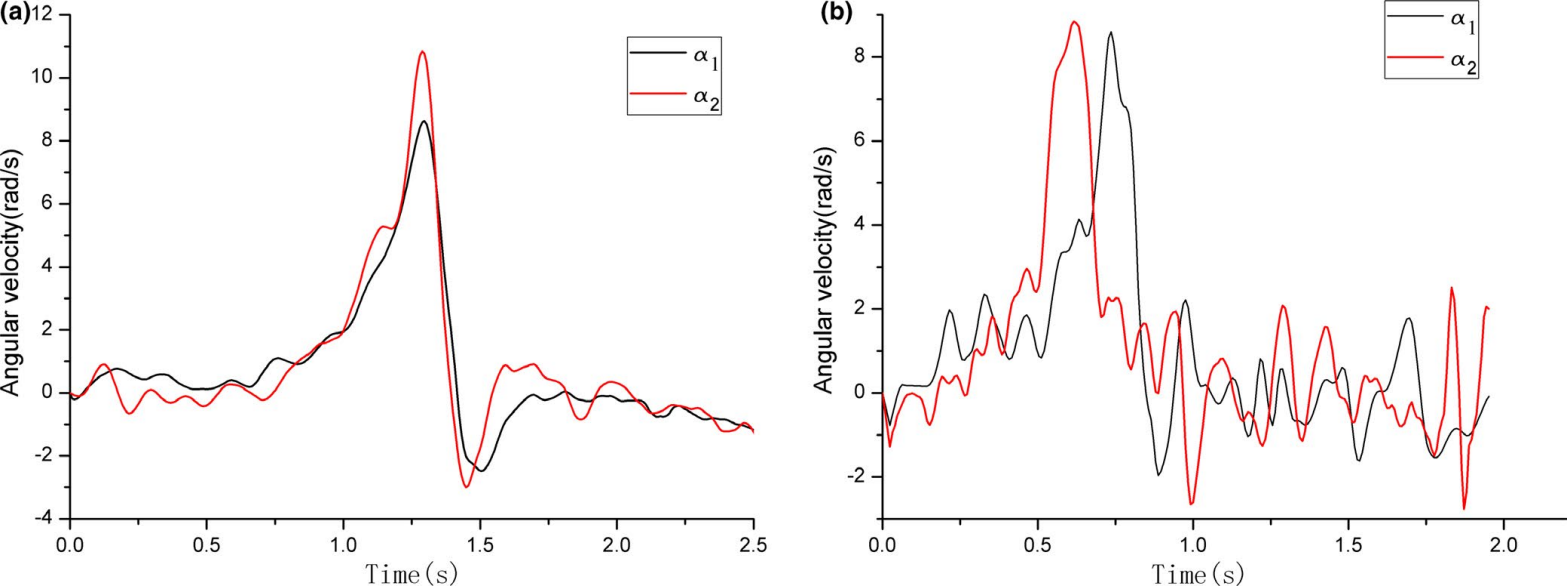
Angular velocity on knee (**a_1_**) and ankle (**a_2_**) of (a) *Rana dybowskii* and (b) *Xenopus laevis*

Additionally, the angular velocity on knee and ankle of two animals was calculated and the representative curves are shown in Figure 7. It can be seen from Table 4 that the maximum angular velocities on knee and ankle of *R. dybowskii* were about 1.5 times higher than that of *X.laevis*. From the curves of angular velocity, it was found that the tendency of angular velocity on knee of *R. dybowskii* was basically same with that of ankle, and maximum values on knee and ankle occurred almost simultaneously. However, there was an obvious time difference between the maximum values of angular velocity on ankle and knee. The rise of angular velocity on ankle of *X.laevis* came earlier than that of knee in takeoff phase.

**TABLE 4:** Angular velocity on knee and ankle (Mean ± SD)

	*R. dybowskii*	*X. laevis*
Maximum angular velocity on knee, (rad/s)	11.062 ± 2.90	6.199 ± 1.388
Maximum angular velocity on ankle, (rad/s)	11.795 ± 2.754	7.748 ± 1.034
Time difference between maximum of knee and ankle, (s)	0.014 ± 0.004	0.122 ± 0.085

## 4 DISCUSSION

According to the experimental results above, a typical short jump of *R.dybowskii* includes four phases: preparation, takeoff, flight, and landing, and the velocity on the hindlimbs of *R. dybowskii* are higher than that of *X.laevis*, besides, the takeoff duration time of *R. dybowskii* is 14.7% shorter than the time used by *X.laevis*.

In order to characterize the kinematics of fore‐ and hind limbs, the rotation range of different joints was examined in this work. The change tendency of knee angle **a_1_** and ankle angle **a_2_** clearly reflects the flexion and extension of knee and ankle, which profiles the importance of knee and ankle in jumping movement. For *R. dybowskii*, **a_1_** and **a_2_** have same tendency, which increase greatly during takeoff phase, and go down in landing phase. This tendency illustrates the hindlimbs' protraction in takeoff and retraction in landing phase. The TMT and midfoot angle have relative small rotation amplitude. The elbow angle and wrist angle could profile the behavior of forelimbs. The trend of elbow angle β_1_ is similar with **a_1_** and **a_2_,** which increases in takeoff and flight phase then declines in landing. The data of wrist angle β_2_ also rise in T2 and falls in T3. The change on β_1_, β_2_ reveals that the *R. dybowskii* would make an important shift to protracting and retracting the forelimbs earlier in takeoff and landing phase. The forelimbs move earlier than the hindlimbs to lift up and control landing, which well cooperated with the motion of hindlimbs. As for *X.laevis,* by contrast, the knee angle **a_1_**, ankle angle **a_2,_** and TMT angle **a_3_** keep rising during the whole jumping process (from T0 to T3), and the midfoot have small rotation range. The elbow tends to protract in T1, while the wrist hardly reveals any regular changes. The forelimbs fail to cooperate with hindlimbs in takeoff, and both forelimbs and hindlimbs are unable to adjust motion timely for landing preparation. The fore‐ and hindlimbs of *X.laevis* show an uncoordinated movement behavior and result in an unsuccessful jump. From observation, it was found that the limbs of both animals did not extend fully straight when impacting the ground. This finding is consistent with the result from previous researches. Actually, it has been proved that frogs would decline the extension of joints before maximum value to prevent the joints damage and at landing to decrease skeletal stress (Azizi & Abbott, 2012; Cox & Gillis Gary, 2015).

Besides, the curve of angular velocity on *R. dybowskii* 's knee is, to a large extent, close to that of ankle, and the maximum value of two joints happens simultaneously. However, the curves of angular velocity of *X.laevis* show the ankle and knee move asynchronously, and the ankle reaches the peak before the knee. From this distinct motion, it is reasonable to infer that the synchronous motion sequence of knee and ankle of *R. dybowskii* could be beneficial to transform angular velocity into forward linear velocity to the most extent; however, the asynchronous behavior of *X.laevi's* hindlimb might be a hindrance for velocity transformation. This motion difference on knee and ankle could be the reason to explain why the kinematic mechanism of *X.laevis* is not suitable for jump on land. On the other side, *X.laevis’* motion mechanism might be more adaptable for swimming than other frogs, which needs to be explored in future work. Additionally, the change of joint angle illustrates the importance of knee and ankle during takeoff. The knee and ankle rotate with large range within one second to produce propulsion, whereas the TMT and midfoot part show limited rotation amplitude.

Based on the experimental result, it can be found that the *R. dybowskii'* limbs have good cooperation in jump behavior. The forelimbs would protract accompanied with the extension of hindlimbs in takeoff phase and retract before the hindlimbs to embrace the ground during landing. Besides, the forelimbs could serve as a pivot during landing, leading the flexed hindlimbs to rotate beneath body and recover the preparation position. The landing sequence of *R. dybowskii* is first hands hit and legs flexing, then the belly hit, legs flexing, and feet moving, which basically follows the "hands‐belly‐feet slap" strategy of *Bombina* and *Lithobates* (Reilly et al., 2016). Nevertheless, the *X.laevis* showed asynchronous and uncoordinated movement on its fore‐ and hindlimbs. Its forelimbs rotate out of the rhythm of hindlimbs' extension in takeoff phase, and both the fore‐ and hindlimbs would not retract to prepare for landing. *X.laevis* land by belly flopping onto the ground and then the limbs rotate to preparation position. The onsets of forelimbs recovery were highly variable in *X.laevis* (thus were not digitized), and this clumsy landing (“belly‐flops”) is close to the *Ascaphus* (Reilly et al., 2016).

## 5 CONCLUSIONS

The present work supplies a specific analysis on kinematic behavior during jumping of two different anuran species, *Rana dybowskii* and *Xenopus laevis*. High‐speed camera and digital tracking technology were used to explore the key features of different joints in limbs during takeoff and landing phase, including velocity, rotation angle. It clarified the main function of forelimbs during jumping, which is not only to lift body in takeoff but also to accommodate the landing posture. Besides, the motion mechanism of limbs during jumping is not stereotyped across species. The fore‐ and hindlimbs of *Rana dybowskii* are much more coordinated than the *Xenopus laevis*, which makes it more adaptable in terrestrial jump.

## CONFLICT OF INTEREST

No competing interests declared.

## AUTHOR CONTRIBUTIONS

Mo Li: Design and draft writing; Zibo Gao, Jili Wang, and Wei Song: Experiment operation and the data analysis; Qingzhu Zhang and Jin Tong: Validation; Lili Ren: Supervision and administration work.

## Data Availability

Data are available from the Dryad Digital Repository. https://doi.org/10.5061/dryad.1ns1rn8t7
